# Causal Effect of Visceral Adipose Tissue Accumulation on the Human Longevity: A Mendelian Randomization Study

**DOI:** 10.3389/fendo.2021.722187

**Published:** 2021-09-01

**Authors:** Bin Yan, Jian Yang, Binbin Zhao, Yanhua Wu, Ling Bai, Xiancang Ma

**Affiliations:** ^1^Department of Clinical Research Center, The First Affiliated Hospital of Xi’an Jiaotong University, Xi’an, China; ^2^Department of Psychiatry, The First Affiliated Hospital of Xi’an Jiaotong University, Xi’an, China; ^3^Department of Cardiology, The First Affiliated Hospital of Xi’an Jiaotong University, Xi’an, China

**Keywords:** visceral adipose tissue, obesity, longevity, causal relationship, Mendelian randomization study

## Abstract

**Objective:**

Observational studies have demonstrated a close relationship between obesity and longevity. The aim of this Mendelian randomization (MR) study is to investigate whether genetic determinants of visceral adipose tissue (VAT) accumulation are causally associated with longevity.

**Methods:**

In this two-sample MR study, we used summary data of genetic determinants (single-nucleotide polymorphisms; p < 5 × 10^−8^) of VAT accumulation based on genome-wide association studies (GWASs). Longevity was defined as an age beyond the 90th or 99th survival percentile. The causal association of VAT accumulation with longevity was estimated with the inverse variance-weighted (IVW) method. Sensitivity analyses, including weighted median, MR-Egger, and MR–pleiotropy residual sum and outlier (PRESSO), were also employed to assess the stability of the IVW results.

**Results:**

Our MR analysis used 221 genetic variants as instrumental variables to explore the causal association between VAT accumulation and longevity. In the standard IVW methods, VAT accumulation (per 1-kg increase) was found to be significantly associated with lower odds of surviving to the 90th (odds ratio [OR] = 0.69; 95% confidence interval [CI] 0.55 to 0.86, p = 8.32 × 10^−4^) and 99th (OR = 0.67; 95% CI 0.49 to 0.91, p = 0.011) percentile ages. These findings remained stable in sensitivity analysis.

**Conclusion:**

This MR analysis identified a causal relationship between genetically determined VAT accumulation and longevity, suggesting that visceral adiposity may have a negative effect on longevity.

## Introduction

Obesity is now a common human health problem, defined as an abnormal or excessive amount of body fat (1, 2). People who consume more calories than spent during their daily activities and exercise are more likely to become obese or overweight (3). However, obesity is also influenced by genetic factors in addition to other environmental influences, including the community, stress, sleep quality, medications, pregnancy, and some diseases (4, 5). Obesity and overweight are typically assessed using anthropometric indicators such as body mass index (BMI), waist circumference, hip circumference, and waist-to-hip ratio (WHR). In addition, obesity is clinically defined according to the measurement of body fat percentage, subcutaneous adipose tissue (SAT), and visceral adipose tissue (VAT). Previous studies also showed that VAT is unique, pathogenic fat depots (6–8).

Several studies have shown that obesity is a risk factor for numerous diseases, including diabetes mellitus, hypertension, coronary artery disease, stroke, fatty liver disease, sleep-disordered breathing, mental health, and cancer (6, 9–11). Obesity has also been shown to be closely related to all-cause mortality (12). Several observational studies revealed that obesity could accelerate the aging process (13, 14). Moreover, a meta-analysis indicated that people with extreme obesity may have a reduced life expectancy by about 14 years (15). However, there is little evidence on the causal relationship between genetic influence of VAT accumulation and longevity.

Mendelian randomization (MR) analysis is a useful approach for estimating the causal relationship between an exposure factor and outcome based on observational data from genome-wide association studies (GWASs) (16). Single-nucleotide polymorphisms (SNPs) typically serve as the instrumental variables for investigating the causal role of an exposure factor on a disease or disease-related outcome. A valid instrumental variable is one that is (1) associated with the exposure, (2) independent of confounders, and (3) independent of the outcome conditional on the exposure and confounders (17). In this two-sample MR analysis, we employed the genetic variants (SNPs) of VAT accumulation as instrumental variables to explore the causal relationship with longevity.

## Materials and Methods

### Study Population

We obtained GWAS summary data for VAT accumulation (4,198 individuals in training datasets and 396,220 individuals in application datasets) derived from a large-scale GWAS (18). The longevity groups were individuals with age beyond the 90th (n = 11,262) or 99th (n = 3,484) survival percentile obtained from GWAS meta-analyses, and the control group (n = 25,483) were individuals whose age at death or at last contact was at or below the 60th survival percentile (19). The details of all datasets used in this MR analysis are summarized in **Supplement Table 1**.

### SNP Selection

**Figure 1:**
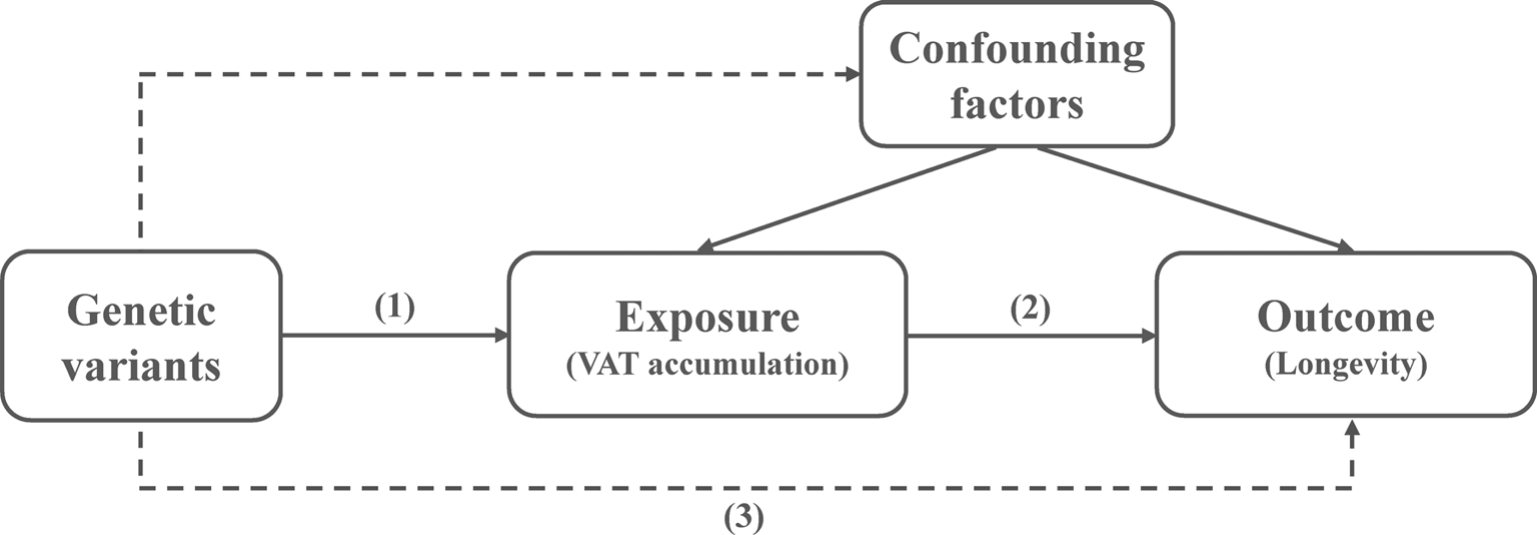
The schematic diagram for the Mendelian randomization study.

We selected SNPs that have been shown to be significantly associated with VAT accumulation (21,727 SNPs) at a genome-wide significance level (p < 5 × 10^−8^), which were also available in the longevity datasets. We further performed linkage disequilibrium analysis using PLINK1.9 based on a threshold of r^2^ < 0.001 in a 10,000-kb window from the 1000 Genomes phase 3 European reference population. The schematic diagram of this two-sample MR study is shown in **Figure 1**.

The strength of the associations of these SNPs with VAT accumulation was measured by R^2^ and *F* statistics. *F* > 10 was recommended for MR analysis, which is calculated as *F* = (N − K − 1)/K × R^2^/(1 − R^2^), where N is the sample size K is the number of SNPs. R^2^ was calculated as 2 **×** MAF **×** (1 − MAF) **×** (beta estimate)^2^, in which MAF is the minor allele frequency (20, 21).

### Statistical Analysis

We utilized 221 genetic variants as instrumental variables to estimate the causal association of VAT accumulation with longevity. In MR study, a valid instrumental variable is one that is 1) associated with the exposure, 2) independent of confounders, and 3) independent of the outcome conditional on the exposure and confounders. Inverse variance-weighted (IVW) approach was used as a standard MR method in our study, which could provide a consistent estimate of the causal effect by combining the ratio estimates of each variant in a fixed-effects meta-analysis model (17). Sensitivity analyses, including weighted median, MR-Egger, and MR–pleiotropy residual sum and outlier (MR-PRESSO), were also performed to test the stability of the IVW results. The weighted median estimator is a method in which at least 50% of the weights are considered valid instrumental variables (22). MR-Egger was further utilized to assess the directional pleiotropy and to provide an estimate of a causal effect, which could test null causal hypothesis and consistent causal effect estimates even if all genetic variants are invalid instrumental variables (based on the assumption that the association of each genetic variant with the exposure is independent of the pleiotropic effect of the variant) (23). MR-PRESSO was employed to detect and correct for horizontal pleiotropic outliers. The MR-PRESSO outlier test requires at least 50% of the variants to be valid instruments and depends on the InSIDE (instrument strength independent of direct effects) conditions (24). A two-sided p-value < 0.05 was considered to indicate a statistically significant association. All steps of the MR analysis were performed in R version 3.6.0 using the R packages MendelianRandomization and MR-PRESSO.

## Results

### Instrumental SNPs

Among all SNPs associated with VAT accumulation in GWAS, 221 independent SNPs (*F* = 69.3) were identified with the lowest p-values, and these instruments explained 3.7% of variance (*F* = 69.3) (**Supplement Tables 2** and**3**).

### VAT Accumulation and Longevity at the 90th Survival Percentile

**Figure 2:**
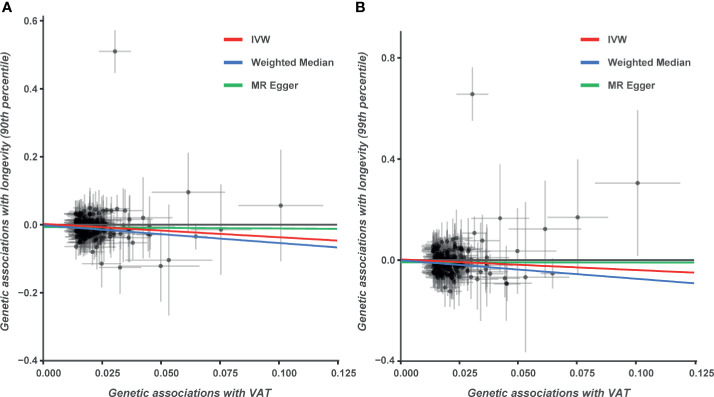
Genetic associations between visceral adipose tissue (VAT) accumulation and longevity. **(A)** Genetic effect of VAT accumulation on survival to the 90th percentile age. **(B)** Genetic effect of VAT accumulation on survival to the 99th percentile age.

VAT accumulation (per 1-kg increase) was significantly associated with lower odds of surviving to the 90th (odds ratio [OR] = 0.69; 95% confidence interval [CI] 0.55 to 0.86, p = 8.32 × 10^−4^) percentile age based on IVW method (**Table 1** and **Figure 2A**). Weighted median (OR = 0.58; 95% CI 0.46 to 0.75, p = 1.94 × 10^−5^) also showed a significant association between VAT accumulation and longevity. However, no significance was found in the MR-Egger test (OR = 0.96; 95% CI 0.48 to 1.91, p = 0.910). This may be due to the fact that MR-Egger has lower precision than IVW method and relies on more power demand (22). In addition, there was no evidence of directional pleiotropy based on the MR-Egger intercept (value: −0.007; p = 0.312). MR-PRESSO identified two outliers (rs2253310 and rs429358). VAT accumulation was still associated with the longevity at the 90th survival percentile after excluding the outliers (OR = 0.65; 95% CI 0.55 to 0.76, p = 1.03 × 10^−7^).

**Table 1:** Mendelian randomization association between genetically determined VAT accumulation and longevity.

Exposures	Methods	SNPs	Longevity (90th percentile)		Longevity (99th percentile)	
			OR (95% CI)	p-Value	OR (95% CI)	p-Value
VAT accumulation		221				
	IVW		0.69 (0.55 to 0.86)	8.32 × 10^−4^	0.67 (0.49 to 0.91)	0.011
	Weighted median		0.58 (0.46 to 0.75)	1.94 × 10^−5^	0.48 (0.32 to 0.71)	2.22 × 10^−4^
	MR-Egger		0.96 (0.48 to 1.91)	0.910	0.99 (0.38 to 2.61)	0.984
	Egger intercept		Value: −0.007; p = 0.312		Value: −0.009; p = 0.407	
	MR-PRESSO		0.65 (0.55 to 0.76)	1.03 × 10^−7^	0.63 (0.50 to 0.81)	2.57 × 10^−4^

MR-PRESSO identified two outliers (rs2253310 and rs429358) for both 90th percentile and 99th percentiles of longevity. Results are presented as OR and 95% CI.

95% CI, 95% confidence interval; IVW, inverse variance weighted; MR-Egger, Mendelian randomization–Egger; MR-PRESSO, Mendelian randomization–pleiotropy residual sum and outlier; OR, odds ratio; SNP, single-nucleotide polymorphism; VAT, visceral adipose tissue.

### VAT Accumulation and Longevity at the 99th Survival Percentile

Similar to the analysis at the 90th survival percentile, we found a significant causal relationship between VAT accumulation and the survival to the 99th percentile age (OR = 0.67; 95% CI 0.49 to 0.91, p = 0.011) in IVW analysis (**Table 1**, **Figure 2B**). This finding was also stable in sensitivity analyses, including weighted median (OR = 0.48; 95% CI 0.32 to 0.71, p = 2.22 × 10^−4^) and MR-PRESSO (OR = 0.63; 95% CI 0.50 to 0.81, p = 2.57 × 10^−4^), but not in the MR-Egger analysis. No directional pleiotropy effects were found based on the MR-Egger intercept (value: −0.009; p = 0.407). We also performed the leave-one-out analysis; the results indicated that the causal effect still existed and even excluded single SNPs one by one (**Supplement Tables 4** and **5**).

## Discussion

In this two-sample MR study, we investigated the causal relationship between genetic determinants (SNPs) of VAT accumulation and longevity, demonstrating a significant association of VAT accumulation with lower odds of surviving to both the 90th and 99th percentile ages. Our MR analysis provides evidence that visceral adiposity may play a negative role in longevity.

Obesity is a common public health condition worldwide (5). Besides, visceral fat is regional fat depots that are located inside the abdominal cavity and stored around the abdominal organs, can be estimated accurately by magnetic resonance imaging, computed tomography, or dual-energy X-ray absorptiometry, and has also been utilized to evaluate visceral fat (25). Previous observational studies revealed that obesity was closely related to all-cause mortality (14, 26, 27). A pooled analysis of 20 prospective studies showed that extreme obesity (BMI 40.0–59.9 kg/m^2^) may shorten life expectancy by up to 14 years (15). In addition, the Global BMI Mortality Collaboration found that both obesity and overweight were significantly associated with higher all-cause mortality based on a meta-analysis of 239 prospective studies in four continents (12). Kuk et al. also found that visceral fat is an independent predictor of all-cause mortality in men. The OR for mortality increased with increasing visceral fat mass and visceral fat area (28). But Britton et al. did not observe a significant association between VAT and all-cause mortality (29). Obesity may reduce the human life span and could affect cellular and molecular processes in a manner resembling the aging process, including telomere attrition, epigenetic alterations, mitochondrial dysfunctions, cellular senescence, stem cell exhaustion, deregulated nutrient sensing, altered intercellular communication, genomic instability, and loss of proteostasis (13). However, there has been minimal evidence about the causal relationship between genetic determinants of VAT accumulation and longevity.

MR approach is a genetic epidemiology method to investigate the causal relationship between exposure factor and outcome based on GWAS data. Previous MR studies have found a causal relationship between BMI and human life span (30). In the present study, we employed MR analysis to investigate the causal role of genetically determined VAT accumulation on longevity. The longevity phenotype was defined as the survival to the age of the top 10% or 1% of survivors in a population (i.e., beyond the age corresponding to the 90th or 99th survival percentile) (19). Using standard MR analysis (IVW), we found a significant association between genetic determinants (SNPs) of VAT accumulation and longevity at both the 90th and 99th percentile ages. Our findings indicated that individuals with visceral adiposity may have shortened human life. The effective interventions were necessary for people with visceral adiposity.

The mechanisms linking VAT accumulation with a short life span is not clear, but some VAT-related biological mechanisms may explain the phenomenon. Compared with SAT adipocytes, VAT adipocytes are prone to be more metabolically active, more sensitive to lipolysis, and more insulin-resistant. Moreover, VAT is found to be associated with greater expression of proinflammatory cytokines and more likely to exhibit endothelial dysfunction than SAT (31, 32). Observational studies were also demonstrated that VAT accumulation was associated with diabetes mellitus and cardiovascular disease. The potential mechanism deserves further study.

This MR study has several strengths. First, MR is an analytic approach using genetic variants as instruments to explore the causal relationship between a modifiable exposure and outcome based on observational data from GWAS. Compared with an observational study, the MR design has more powerful control over reverse causation and confounding variables. Second, we utilized the weighted median, MR-Egger, and MR-PRESSO to examine the stability of the IVW results. Finally, this is the first MR study to investigate the causal relationship between genetically determined VAT accumulation and longevity. There are also several limitations of our study that should be acknowledged for interpretation of these results. Multiple sensitivity analyses were performed to test and correct for horizontal pleiotropy, but the potential pleiotropy bias could not be fully eliminated. In addition, the populations were based on European ancestry; therefore, our results cannot be generalized to all ethnic groups.

In conclusion, we identified a significant causal relationship between VAT accumulation and longevity. The findings indicate the need for effective interventions for visceral adiposity to prevent the impact on life span.

## Data Availability Statement

The datasets presented in this study can be found in online repositories. The names of the repository/repositories and accession number(s) can be found in the article/**Supplementary Material**.

## Author Contributions

BY and XM raised the idea for the study. BY, JY, BZ, YW, XM, and LB contributed to the study design, and the writing and review of the report. BY and JY did the data analysis. XM handled supervision in our study. All authors contributed to the article and approved the submitted version.

## Funding

This study was supported by the Natural Science Basic Research Program of Shaanxi (No. 2021JQ-395) and the Clinical Research Award of The First Affiliated Hospital of Xi’an Jiaotong University, China (No. XJTU1AF-CRF-2019-022).

## Conflict of Interest

The authors declare that the research was conducted in the absence of any commercial or financial relationships that could be construed as a potential conflict of interest.

## Publisher’s Note

All claims expressed in this article are solely those of the authors and do not necessarily represent those of their affiliated organizations, or those of the publisher, the editors and the reviewers. Any product that may be evaluated in this article, or claim that may be made by its manufacturer, is not guaranteed or endorsed by the publisher.
